# Short-Term Effects of Spirulina Consumption on Glycemic Responses and Blood Pressure in Healthy Young Adults: Results from Two Randomized Clinical Trials

**DOI:** 10.3390/metabo12121180

**Published:** 2022-11-25

**Authors:** Foteini Lympaki, Marianna Giannoglou, Emmanuella Magriplis, Dionysia Lydia Bothou, Varvara Andreou, George D. Dimitriadis, Giorgos Markou, Antonis Zampelas, Georgios Theodorou, George Katsaros, Emilia Papakonstantinou

**Affiliations:** 1 Laboratory of Dietetics and Quality of Life, Department of Food Science and Human Nutrition, School of Food and Nutritional Sciences, Agricultural University of Athens, 75 Iera Odos, 11855 Athens, Greece; 2 Institute of Technology of Agricultural Products, Hellenic Agricultural Organization “DEMETER”, 14123 Lykovrisi, Greece; 3 Sector of Medicine, Medical School, National and Kapodistrian University of Athens, 15772 Athens, Greece; 4 Department of Animal Science, Agricultural University of Athens, 75 Iera Odos, 11855 Athens, Greece

**Keywords:** spirulina, cookies, dose, glycemic index, glycemic response, blood pressure, appetite, healthy adults

## Abstract

The effects of spirulina consumption added in foods were investigated in two crossover clinical trials (*n* = 13 different healthy adults). In Trial-1 adults consumed cookies with-and-without spirulina (3.12 g per 100 g final product; 2.5 g spirulina per 50 g available carbohydrates) according to glycemic index (GI) methodology. In Trial-2, adults consumed 4 g, 6 g, and 8 g spirulina as beverage diluted in 50 g D-glucose vs. 50 g plain D-glucose. Capillary blood glucose samples were collected at 0, 15, 30, 45, 60, 90, and 120 min and blood pressure (BP) was measured at beginning and end of each visit in both trials. Trial-1: both cookies with and without spirulina provided medium GI values (59 and 60, respectively, on glucose-scale), but no significant differences were found for BP. Trial-2: both 4 g and 8 g spirulina lowered postprandial glucose at 120 min (95% CI: −1.64 to −16.12 and −1.23 to −15.87, respectively). The results explained 29% of variation. Only 8 g spirulina decreased significantly 90–120 min area under the curve (AUC) for glucose and systolic BP (−4%). No differences were found for fasting glucose. Adding spirulina to cookies did not affect glucose responses and BP. Only 8 g provided significantly lower 90–120 min-AUC for glucose and BP compared to 4 g, 6 g-and-D-glucose, indicating advantages to glycemic control and hypertension.

## 1. Introduction

Microalgae are photosynthetic organisms that grow in various aquatic habitats in a range of conditions [1]. Specific species of microalgae are known to be used in human nutrition for years due to their high nutritional value [1]. Spirulina (*Arthrospira platensis* and *Arthrospira maxima*) is a blue-green, filamentous, sustainable, and ecofriendly cyanobacterium [1]. It contains high amounts of protein (60–70% of its dry weight), 4–7% lipids, and 13.5% carbohydrates [1]. It also contains, among others, B vitamins, iron, calcium, and antioxidants [1]. The Food and Drug Administration (FDA) approved spirulina as GRAS (Generally Recognized as Safe) food without toxicological effects on human health [2]. The main pigment-protein complexes of spirulina are C-phycocyanin and allophycocyanin [2]. According to the World Health Organization (WHO)/Food and Agriculture Organization of the United Nations (FAO) the total dietary exposure of spirulina from use as a food ingredient is estimated at 2000 g/person per day, expressed as phycocyanin, and 2–10 g/day of spirulina taken as dietary supplement [3]. Few studies have examined the addition of spirulina to foods. The results from a study examining the addition of spirulina flour to the dough of durum wheat semolina spaghetti at two different concentrations, 5% and 10%, reported: a significant increase in protein and dietary fiber content; a spirulina dose-dependent decline of the in vitro starch digestibility; but also a dose-dependent decline of pasta overall sensory quality [4]. Another study reported that adding spirulina (0.5, 1.0, and 2.0 g/100 g DW) to semolina flour produced spaghetti richer than the control in fatty acids, and enhanced the nutritional and sensorial quality of pasta, although firmness was positively affected by spirulina inclusion [5]. Similar results were reported with handmade ice cream containing microencapsulated spirulina (1% biomass in relation to ice cream mass), producing ice cream with 35% to 53% more protein [6]. 

The glycemic index (GI) is a tool developed to classify carbohydrate containing foods according to their time integrated effects on postprandial glycemia [7]. The GI depicts both the standardized and relative postprandial glucose response based on an equal amount of available carbohydrate and relative to a referent food [7]. Foods containing carbohydrate that is digested, absorbed, and metabolized quickly are considered high GI foods (GI ≥ 70 on glucose scale); whereas those that are digested, absorbed, and metabolized slowly are considered low GI foods (GI ≤ 55 on glucose scale) [7]. The consumption of high GI foods is associated with increased chronic disease risk [8,9,10]; whereas low to medium GI foods is associated with lower chronic disease risk [11]. Results from one study examining the acute effects of adding 2.5 g spirulina in cereal bars, compared to conventional and cereals bars with brewers spent grain and dehydrated apple, reported lower blood glucose peaks after spirulina consumption, but a similar and gradual release of glucose in blood between the test cereals under study [12]. Another study examining the effects of four Indian regional meals all supplemented with 2.5 g of spray dried spirulina powder in healthy adults, reported inconclusive effects of spirulina on postprandial glycemia [13].

Results from a systematic review assessing the effects of spirulina supplementation on metabolic syndrome components, hepatic, and related inflammatory markers from 22 randomized clinical trials (RCTs), reported that oral dosage ranging from 1–19 g/day for 0.5–6 months had positive effects on metabolic syndrome components, although determining the optimal dosage still needs further investigation [14]. Regarding the effects of spirulina supplementation on blood pressure (BP), the results from RCTs in healthy individuals, overweight or obese elderly, people with type 2 diabetes, and people with non-alcoholic fatty liver disease (NAFLD), short-term (few hours) or longer-term (up to 3 months), were inconclusive and the optimal dose is still unknown [15,16,17,18,19,20,21]. Spirulina supplementation for 3 months has also been reported to reduce appetite [22,23].

Based on the literature, a dose of 2.5 g spirulina added in foods showed some promising effects on health indices. Therefore, in order to evaluate the short-term effects of spirulina consumption from foods on postprandial glycemic responses, BP, and subjective appetite, in metabolically healthy adults, two cross-over, randomized clinical trials were carried out: (a) to examine the effects of added spirulina in cookies; and (b) to examine the effects of three different doses of spirulina consumed as glucose beverages in the aforementioned health indices.

## 2. Materials and Methods

### 2.1. Participants

Healthy men and women, aged 18 to 55 years, were recruited in these two randomized, crossover, clinical trials by a variety of methods, including online advertisements, notices posted around the university campus, and leaflets. The sample size calculation for both studies used the *t* distribution assuming an average coefficient of within-individual variation (CV) of the incremental area under the curve (iAUC) values of 25%. Ten participants led to 80% power to detect a 33% between individual difference in iAUC with two-tailed *p* < 0.05. In both investigations we enrolled and studied 13 participants, accounting for potential losses due to lack of compliance. Volunteers underwent an initial screening, including anthropometry measurements (height, body weight, waist, and hip circumferences) and body composition analysis via bioimpedance method (InBody 230, Biospace, Urbandale, IA, USA). Fasting blood glucose was measured in the initial screening via finger prick (calibrated MediSmart Ruby glucose meter with lancing device, Lilly-Pharmaserv SA, Athens, Greece). In addition, participants completed a questionnaire on general health. Participants were nonsmokers, had a healthy body mass index (BMI between 18.5 and 24.9 kg/m^2^), normal fasting blood glucose values (<100 mg/dL), normal blood pressure (Omron, Intellisense, HEM-907, Omron Hellas, Athens, Greece; systolic blood pressure (SBP) < 120 mmHg and diastolic blood pressure (DBP) < 80 mmHg), and no medical conditions, such as cardiovascular diseases, diabetes mellitus, liver or kidney diseases, gastrointestinal disorders, nor taking medications known to affect glycemia, such as metformin, glucocorticoids, and thiazide diuretics. Female participants were not pregnant/lactating nor were they diagnosed with polycystic ovary syndrome. All 13 participants in both trials completed all treatments and were included for analyses.

Both studies were conducted at the Laboratory of Dietetics and Quality of Life, Agricultural University of Athens, Greece. All subjects gave their informed consent for inclusion before participating in the studies. Both studies were conducted in accordance with the Declaration of Helsinki, and the protocols were approved by the Bioethics Committee of the Agricultural University of Athens (EIDE Reference Number: 50/ 12/10/2021 and 54/ 06.06.2022, respectively). The trials were registered at Clinicaltrials.gov (NCT05484323 and NCT05484336).

### 2.2. Study Design

**Figure 1:**
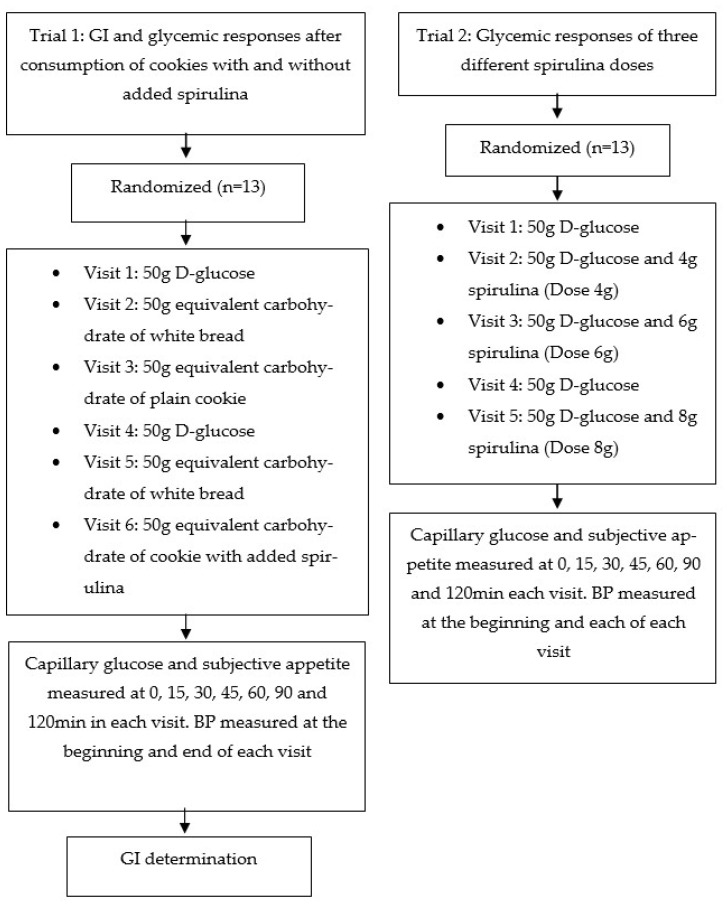
Flowchart of the two current investigations given as examples of the crossover, randomized procedure. Eligible participants were studied in separate days over a period of 3–9 weeks with an interval of no less than 40 h and more than 2 weeks between tests. Participants attended the sessions of around 3 h duration, separated by a wash-out period of at least two days. Abbreviations: GI = glycemic index; BP = blood pressure.

The studies’ design is described in Figure 1.

#### 2.2.1. Trial 1

In this first pilot study, two types of handmade cookies, a) without addition of spirulina and b) with the addition of 3.12 g spirulina per 100 g of ready to eat product (4.5% by DW) (approximately 0.47 g added spirulina per cookie; producing 2.5 g spirulina in cookies containing 50 g available carbohydrates), were prepared at the Institute of Technology of Agricultural Products, Hellenic Agricultural Organization “DEMETER”. The amount of spirulina content was selected based on an overall organoleptic evaluation of cookies containing different doses of spirulina by a semi-trained panel of 15 people. Spirulina content higher than 2.5 g was scored below the acceptable limit. Consequently, a clinical trial was performed in order to confirm whether cookies containing 50 g available carbohydrates, would produce acute clinically significant differences in postprandial glycemia within 120 min. The GIs of both cookies were evaluated. The GI was determined according to ISO 26642:2010 International Organization for Standardization method and procedures [7]. The trial consisted of six dietary treatments in a randomized, blind, cross-over design: two glucose reference drinks, white bread tested twice, and the cookies without spirulina (C) and cookies with added spirulina (CS) tested once (Figure 1). For the simple randomization of the sequence of the tested foods the online computer software (Social Psychology Network, Middletowin, CT, USA) was used (http://www.randomizer.org; Accessed on 1 March 2021) [24]. Two researchers not involved in the collection and analysis of the scientific data, were responsible for the randomization of the volunteers to the intervention days examining the test foods and the double-blind condition. Cookies differed slightly in color and texture, and thus, only open-label type conditions were achieved; both the researchers collecting the data and the participants consuming the test foods were aware of the test food type provided.

Volunteers arrived at the Lab of Dietetics and Quality of Life at 8:45–9:00 h in the morning following an overnight fast of 10–14 h in both Trials 1 and 2. Volunteers were asked to maintain stable dietary and activity/exercise habits throughout their participation in both investigations. Participants were also instructed to refrain from alcohol on the previous evening, from vigorous exercise on the morning of the test, and were only allowed to eat the provided foods throughout the test sessions in both Trials 1 and 2. Compliance to the above-mentioned instructions were monitored in every visit with a 24-h dietary recall and with the International Physical Activity Questionnaire. In the case that a participant was not feeling well or had not complied with the preceding experimental conditions, the test was not carried out and was rescheduled for another day. On each test occasion volunteers were weighed, to assure weight stability in both Trials 1 and 2. In both Trials 1 and 2, each session consisted of a test food that had to be consumed at a comfortable pace within 10–15 min, and 2 h post-meal measurement of metabolic blood parameters.

In Trial 1, participants received, in random order, the reference foods (D-glucose), tested two times, the white bread, tested two times, and the two test cookies, C and CS, tested once, in different weeks, with random sequence in accordance with the recommended GI methodology (Figure 1) [8]. All the test foods and the reference food were given in portions containing 50 g available carbohydrates (50 g D-glucose, 91.40 g white bread, 76.88 C, and 80.16 g CS). Test foods were served with 250 mL water as a drink in all six sessions.

The available carbohydrates were determined using the Megazyme assay kit (Megazyme kit-K-ACHDF, Megazyme Ltd., Scotland, UK), which calculates only the carbohydrates that can be absorbed (sugars and digestible starch), neglecting dietary fiber and resistant starch. The nutritional characteristics of the studied test cookies are described in Table 1 and the ingredients of the cookies in Table 2. Table 3 shows the nutritional composition of spirulina powder that was used in both studies.

#### 2.2.2. Trial 2

A second pilot trial was designed to test three different doses of spirulina consumption on postprandial glycemic responses. The study consisted of five dietary treatments in a randomized, double-blind, cross-over design on 13 different volunteers from Trial 1: two glucose reference beverages (50 g D-glucose), one beverage with 50 g D-glucose and 4 g spirulina (Dose 4 g), one beverage with 50 g D-glucose and 6 g spirulina (Dose 6 g), and one beverage with 50 g D-glucose and 8 g spirulina (Dose 8 g) (Figure 1). The above doses of spirulina were diluted in 250 mL of water. For the simple randomization of the sequence of the foods the online computer software (Social Psychology Network, Middletown, CT, USA) was used (http://www.randomizer.org; Accessed on 1 March 2021) [24]. As in Trial 1, two researchers not involved in the collection and analysis of the scientific data, were responsible for the randomization of the volunteers to the intervention days examining the test foods and the double-blind condition. All beverages were served in dark paper cups covered with a lid to achieve double blind conditions, where nor the researchers collecting the data, nor the participants consuming the test beverages, were aware of the test food type provided. In Trial 2, participants received in random order the reference foods (D-glucose), tested two times and the three test beverage spirulina doses, dose 4 g, dose 6 g, and dose 8 g, tested once, in different weeks, with random sequence (Figure 1), following a protocol for testing glycemic responses according to Brouns et al., (2005) [25]. All the test beverages and the reference food were given in portions containing 50 g available carbohydrates.

### 2.3. Blood Glucose Concentrations

On each test occasion in both trials two fasting blood samples were obtained by finger-stick at 5 min intervals (−5, 0); the average of the glucose concentrations at these two time points was taken to be the baseline (fasting) concentration. Participants were then served the test food. Further finger-prick blood glucose samples were collected at 15, 30, 45, 60, 90, and 120 min after starting to eat. Each blood glucose time value was the mean of two blood samples from the same drop of blood of each participant. Before and during a test, a blood glucose test record was filled out with the participant’s initials, identification number, date, body weight, test food, beverage, time of starting to eat, time it took to eat, time and composition of last meal, and any unusual activities. During the 2 h test, volunteers remained seated quietly. After the last blood sample had been obtained volunteers were offered a snack and were informed that the session was completed.

**Figure 2:**
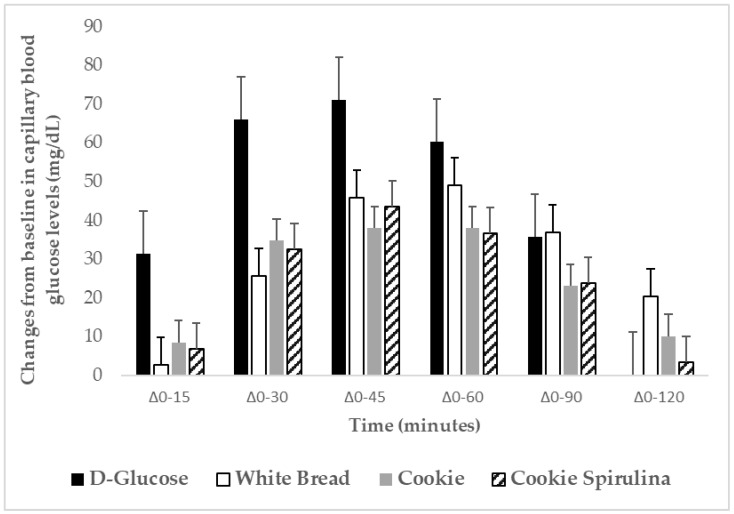
Changes from baseline (Δ) for capillary blood glucose concentrations (mg/dL) after the consumption of the test foods: plain cookies containing 50 g available carbohydrates, cookies containing 50 g available carbohydrates (2.5 g spirulina), white bread, and the reference food (D-glucose) (*n* = 13). Data are means ± SEM. Data were compared by post hoc analysis of repeated measures ANOVA. The models included the factors “subject” (id), “sequence” for inter-subject variation, and “period” and “treatments” to account for intra-subject variability.

To standardize all data collection procedures, capillary blood glucose monitoring was performed at the fingertip (distal phalange of the third finger). Blood glucose was measured with calibrated glucometers using glucose dehydrogenase-FAD test strips (Ruby blood glucose test strips, Lilly-pharmaserv S.A., Athens, Greece), which show no reactivity to any sugars other than glucose and have better heat resistance and oxygen resistance. The repeatability and within laboratory coefficient variations were 3.3%. The average blood glucose response curve was plotted by calculating the mean blood glucose concentrations of all participants at each time point (Figure 2). Then, for each sample and each study participants, the incremental area under the curve (iAUC) was calculated geometrically, using the trapezoid rule, and ignoring the area beneath the baseline [8]. The GI calculation in Trial 1 for each test food sample used the method referred to as the mean of the ratios. For each participant, the ratio between the individual iAUC after consuming the test food sample and the iAUC for the same participant after consuming the reference food was calculated and expressed as a percentage value. Then, the GI of each test food was calculated as the average value of the ratios across all the participants consuming the test food samples. Peak blood glucose, defined as the highest recorded blood glucose value minus the baseline value, and peak blood glucose time, defined as the time elapsing from the start of a meal to the highest recorded blood glucose value, were calculated in both trials.

### 2.4. Blood Pressure (BP)

In both Trials 1 and 2, BP (SBP and DBP) was measured at the screening stage and at the beginning and end of each test food session using an upper arm digital BP monitor (Omron HEM-907, Omron Hellas, Athens, Greece) in a quiet, warm setting in both trials. Participants were rested for 5 min in the supine position with their arm supported at the level of the heart after which three BP measurements were taken by an already introduced member of our trained research team to avoid the “white coat effect”, at 1 min intervals, with the three readings averaged.

### 2.5. Subjective Appetite Ratings

In both Trials 1 and 2, participants rated their hunger, desire to eat, and perceived fullness after eating on 100 mm line visual analogue scales (VAS), ranging from not at all (0 mm) to extremely (100 mm), with for example neither hungry (0 mm), full (100 mm), or having desire for food in the middle (50 mm). VAS were given in the form of a booklet, one scale per page [26]. VAS ratings were obtained at times 0, 15, 30, 45, 60, 90, and 120 min post-test meal consumption.

### 2.6. Dietary Intake

Dietary intake was assessed by 24-h recalls at every visit in both Trials 1 and 2 by a trained nutritionist, member of the research team, and analyzed using the Diet Analysis Plus program, as well as using Hellenic and European Food Composition Databases (http://www.eurofir.org/foodinformation/foodcomposition-databases-2/; Accessed on 1 March 2021). The databases were modified to include new foods and recipes. The purpose of collecting dietary intake was to confirm that participants refrained from changing their eating habits until the study was completed.

### 2.7. Statistical Analysis

Data were entered into a spreadsheet by two different individuals and the values compared to assure accurate transcription. Incremental area under the blood glucose curves in both Trials 1 and 2 (iAUC), ignoring area below fasting, were calculated. For the purposes of AUC calculation, fasting glucose was taken in both trials to be the mean of the first measurement of the blood glucose concentrations at times −5 min and 0 min. The GI in Trial 1 was calculated by expressing each participant’s iAUC for the test food as a percentage of the same participant’s mean iAUC for the two D-glucose drinks controls. If values were found to have >2 SD above the mean, they would be excluded. No outlying GI values were found. The ISO method requires, for a valid GI measurement, that the mean within-individual coefficient of variation of glycemic responses elicited by repeated tests of oral glucose (termed reference CV) is ≤30% [8]. We calculated reference CV for blood glucose according to the ISO method; namely the mean, SD and CV (100 × SD/mean) of the glucose iAUC values elicited by the two repeated tests of 50 g glucose were calculated for each participant and the mean of the resulting values was the reference CV. The blood glucose concentrations in both Trials 1 and 2, at each time, AUC, GI values, subjective appetite, and BP were subjected to repeated-measures ANOVA examining for the main effects of test food and the food × participant interaction

Data are presented as mean ± standard error of the mean (SEM), unless otherwise specified. Statistical tests for data analysis were performed according to data distribution; tested by P-P and kernel density plots. Baseline differences for normally distributed continuous variables were evaluated using analysis of variance (ANOVA), and Kruskal-Wallis test was used for skewed continuous data. Pearson’s chi square test was performed to determine between group differences for categorical variables. Glycemic load (GL) in Trial 1 was calculated using the formula: GL = GI × g of available carbohydrate in a typical food serving/100.

Between treatment, analysis of variance (ANOVA) for a 2 × 2 cross-over study was conducted for blood glucose in both trials and salivary insulin in Trial 1, assuming as per data results a fixed error. In a 2 × 2 design we assume that there are no individual effects since a complete randomization process was followed for treatment allocation. The models included the factors “subject” (id), “sequence” for inter-subject variation, and “period” and “treatments” to account for intra-participant variability. Time × test food interaction was evaluated. Multiple comparisons between the interventions were tested post hoc using the Tukey test with Bonferroni correction. For all other parameters, one-way ANOVA was used to investigate differences between test foods followed by post hoc Tukey test and Bonferroni correction.

The evaluation of spirulina dose in Trial 2 on postprandial glycemia was explored using a multi-level random effects regression model, since a large between individual variation for same test meals was derived. The model was used to evaluate the blood glucose concentrations (calculated by subtracting blood glucose concentrations at each time increment from the blood glucose baseline values), as well as the AUCs for blood glucose per period that capillary blood samples were collected. Means differing by more than the LSD (least significant difference) were statistically significant, two-tailed *p* < 0.05. Data was analyzed using the SPSS 20.0 software (SPSS Inc., Chicago, IL, USA).

## 3. Results

### 3.1. Trial 1

#### 3.1.1. Participants’ Baseline Characteristics

The participants’ characteristics are shown in Table 4. There were no intermittent missing values or dropouts.

#### 3.1.2. GI οf Test Foods

According to the ISO 26642:2010 International Organization for Standardization methodology [7], individual GI values were tested for outliers. One value was found to be higher than 2 standard deviation points and was excluded from further analyses. The results of GI and GL for C and CS are presented in Table 5. The results revealed that both C and CS were classified as medium GI (GI > 55 but <70 on glucose scale) foods. Compared to the reference food (D-glucose), all three test foods white bread, C, and CS had significantly lower GI value (Table 5). Both C and CS had significantly lower GL value compared to white bread and the reference food D-glucose (Table 5).

#### 3.1.3. Blood Glucose Concentrations Trial 1

Figure 2 describes the changes from baseline for capillary blood glucose concentrations (mg/dL) after consumption of the test foods and reference food. No significant differences were observed on fasting blood glucose values between D-glucose and test foods (*p* for all >0.05). There was a significant blood glucose × time × test meal interaction (*p* < 0.001) and a main effect of test food on blood glucose concentrations (*p* = 0.006). Compared to the reference food D-glucose, lower blood glucose concentrations were observed as changes from baseline after the consumption of white bread at 15, 30, and 120 min (*p* < 0.001, *p* = 0.001, and *p* = 0.014, respectively). Compared to the reference food D-glucose, lower blood glucose concentrations were observed as changes from baseline after the consumption of CS at 15, 30, 45, and 60 min (*p* < 0.001, *p* = 0.001, *p* = 0.034, and *p* = 0.039, respectively). Compared to the reference food D-glucose, lower blood glucose concentrations were observed as changes from baseline after the consumption of C at 15, 30, 45, 60, and 90 min (*p* < 0.001, *p* = 0.001, *p* = 0.004, *p* = 0.035, and *p* = 0.049, respectively), Compared to the reference food D-glucose, lower peak for blood glucose values were observed as changes from baseline after the consumption of CS and C (*p* = 0.001 and *p* = 0.001, respectively).

There was a main effect of test meal on iAUC for blood glucose (*p* < 0.001). The average intra-participant coefficient variation of iAUC values after the two repeated D-glucose tests was 29%. The 0–120 min iAUC for blood glucose values calculated as changes from baseline for CS and C were significantly lower than those of the reference food D-glucose (*p* = 0.004 and *p* = 0.03, respectively; Table 5).

#### 3.1.4. BP Trial 1

No differences were observed for BP measurements (SBP and DBP) between and among the test foods, white bread, CS, and C, and compared to the reference food D-glucose at all time points (*p* for all > 0.05; data not shown).

#### 3.1.5. Subjective Appetite Ratings Trial 1

No differences were observed for subjective appetite ratings between and among the test foods, white bread, CS, and C, and compared to the reference food D-glucose at all time points (*p* for all > 0.05; data not shown).

### 3.2. Trial 2

#### 3.2.1. Participants’ Baseline Characteristics

The participants’ characteristics are shown in Table 4. There were no intermittent missing values or dropouts.

#### 3.2.2. Blood Glucose Concentrations Trial 2

**Figure 3:**
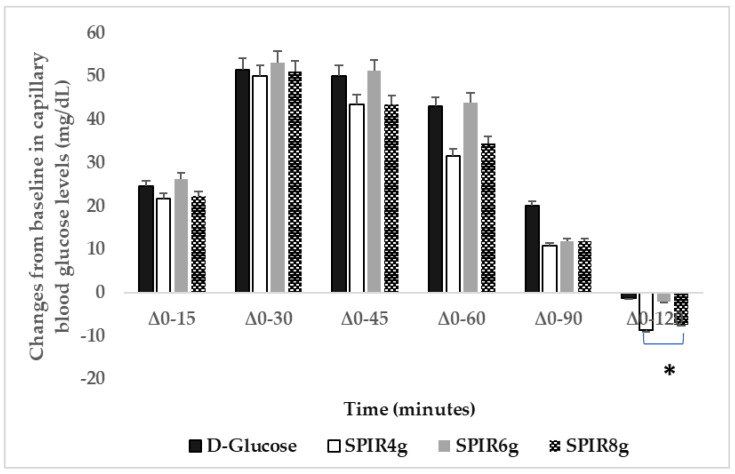
Changes from baseline (Δ) for capillary blood glucose concentrations (mg/dL) after the consumption of test beverages, 50 g D-glucose with 4 g spirulina, 50 g D-glucose with 6 g spirulina, 50 g D-glucose with 8 g spirulina, and the reference beverage (50 g D-glucose) (*n* = 13). Data are means ± SEM. The asterisk denotes a statistically significant difference at the 0.05 level, as derived from repeated measures ANOVA followed by post hoc Tukey’s test. The models included the factors “subject” (id), “sequence” for inter-subject variation, and “period” and “treatments” to account for intra-subject variability. Between group effects were explored using multi-level random effects regression model, clustered by d and adjusted for time. Abbreviation: SPIR = spirulina.

Figure 3 describes the changes from baseline (Δ) for capillary blood glucose concentrations after the consumption of the test spirulina-D-glucose beverages and the reference food D-glucose. No significant differences were observed on fasting blood glucose values between D-glucose and test spirulina doses (*p* for all > 0.05). Between group effects were explored using multi-level random effects regression model, clustered by d and adjusted for time (Table 6). A significant difference was found for Δ0–120 levels for the doses of 4 g of spirulina and 8 g of spirulina consumption (Figure 3). In both cases, the difference was approximately −9 mg/dL for postprandial blood glucose concentrations (95% CI: −1.64 to −16.12 and −1.23 to −15.87, respectively). In no treatments, sequence was time significant (*p* < 0.05). The results explained 29% of the variation. The unobserved heterogeneity within an individual was accounted for by clustering analysis by participant ID number. When the area under the curve (AUC) for blood glucose concentrations for the period of 90–120 min was examined, only the dose of 8 g spirulina had significantly lower values (Table 7). No significant differences were observed as absolute values for 0–120 min incremental area under the curve (iAUC) for blood glucose, peak blood glucose or time to peak for blood glucose (*p* for all >0.05).

#### 3.2.3. BP Trial 2

**Figure 4:**
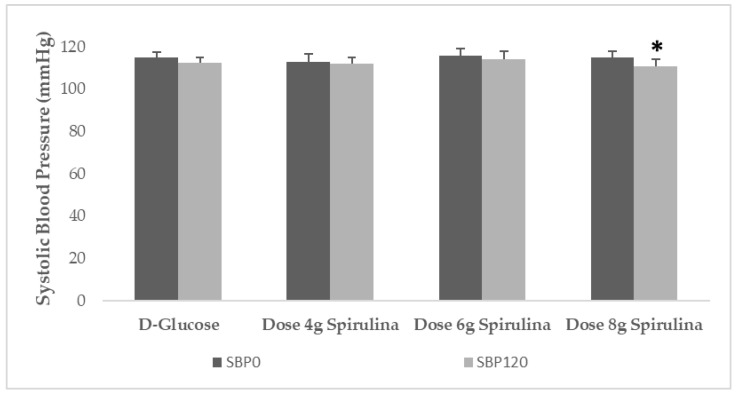
Systolic blood pressure (SBP) at baseline and 120 min post-test beverage consumption. Beverages tested were 50 g D-glucose with 4 g spirulina, 50 g D-glucose with 6 g spirulina, 50 g D-glucose with 8 g spirulina, and the reference test beverage 50 g D-glucose (*n* = 13). Data are means ± SEM. Data were compared by repeated measures analysis using post hoc Tukey test with Bonferroni correction. * = *p* < 0.05. Abbreviations: SBP0: systolic BP at baseline. SBP120: systolic BP at 120 min post-test beverage.

There was a significant main effect of test spirulina beverage on SBP (*p* = 0.012). The consumption of the beverage containing 8 g spirulina showed a significant reduction at 120 min posttest meal in SBP of 4.2 mmHg or 3.7% (*p* = 0.025) (Figure 4). No significant changes from baseline were observed in SBP after the consumption of the other test beverages (*p* for all > 0.05). No differences were observed in DBP between and among test beverages.

#### 3.2.4. Subjective Appetite Ratings Trial 2

No differences were observed for subjective appetite ratings between and among the test beverages, 50 g D-glucose, 50 g D-glucose with 4 g spirulina, 50 g D-glucose with 6 g spirulina, and 50 g D-glucose with 8 g spirulina at all time points (*p* for all >0.05; data not shown).

## 4. Discussion

Spirulina is a functional ingredient and has gained significant scientific interest in the past few years. To the best of our knowledge, our two current investigations examined for the first time: (a) the effects of adding 3.12 g of spirulina per 100 g to cookies, thus examining the dose of 2.5 g spirulina per 50 g available carbohydrates on the GI, following the ISO 26642:2010 International Organization for Standardization [7] method and procedures, and on the glycemic responses; and (b) the effects of three doses of spirulina, namely 4 g, 6 g, and 8 g, added in glucose beverages containing 50 g D-glucose on the postprandial glycemic responses in young healthy adults. The main findings of the two trials were that: (a) contrary to our first hypothesis, adding spirulina to cookies did not produce lower GI or ameliorated postprandial glycemic responses compared to plain cookies prepared with exactly the same recipe and containing the same amount and type of carbohydrates, although both cookies produced medium GI values and significantly lower postprandial blood glucose levels compared to the reference food D-glucose; and (b) in the second investigation, we found that only the glucose beverage containing 8 g of spirulina lowered significantly postprandial glucose concentrations at 90 to 120 min posttest beverage consumption of approximately −9 mg/dL compared to D-glucose, 4 g, and 6 g spirulina. Moreover, only the glucose beverage containing 8 g spirulina decreased significantly SBP by 4.2 mmHg or approximately 4% at 120 min posttest beverage consumption in normotensive healthy young adults.

Hyperglycemia and hypertension are key risk factors for the development of atherosclerotic cardiovascular diseases (CVD) in humans [27]. Therefore, the assessment of glycemic and BP effects of functional foods or ingredients, such as spirulina, may be considered a crucial element for an effective primary and secondary CVD prevention. It has been suggested that a dose of 2–14 g of spirulina supplementation is a safe approach as complementary treatment for chronic diseases [28]. Adding spirulina to foods, such as pasta, at three levels, 5, 10, and 20 g/100 g, was reported to provide high amounts of proteins and phenolic compounds with antioxidant properties, without producing significant changes in estimated in vitro GI values of the tested pasta products, compared to conventional ones [29], which is in partial agreement to findings from our first trial. Two studies examining the addition of 2.5 g spray dried spirulina powder to five Indian rice recipes and four Indian regional meals on glycemic responses of healthy participants, reported varying GIs of the tested meals and claimed potential spirulina induced blood glucose lowering effects when compared to the reference food D-glucose [13,30], which is also in partial agreement with our first trial.

Regarding results from our second current investigation reporting that the dose of 8 g of spirulina consumption as beverage decreased significantly more postprandial glycemic responses acutely, compared to the other doses, 2.5 g in cookies, 4 g as beverage, and 6 g as beverage, adds value to the limited randomized clinical trials (RCTs) literature. However, the optimal dose of spirulina consumption is still unknown, and the results from the few available RCTs are contradictory. The results from three available studies examining the effects of 8 g spirulina supplementation for 12 weeks reported contradictory results, with two studies showing no changes in fasting blood glucose in elderly and people with type 2 diabetes [15,16], and no changes in fasting insulin levels and HbA1c in people with type 2 diabetes [16]; one study showed decreased fasting blood glucose levels in people with type 2 diabetes after spirulina supplementation [31]. The results from the only one available study examining the effects of 6 g spirulina supplementation for 6 months in people with NAFLD showed a significant ~20% reduction in insulin resistance after spirulina supplementation [17]. The results from two studies examining the effects of 4–4.5 g spirulina supplementation from 6 to 12 weeks in people with overweight or obesity, or with type 2 diabetes, showed no changes in fasting blood glucose or insulin levels after spirulina supplementation [19,32]. The results from four available studies examining the effects of 2 g spirulina supplementation from 8 to 12 weeks in overweight or obese individuals, or in people with type 2 diabetes or with NAFLD were also contradictory, with two studies reporting decreased insulin resistance in overweight or obese individuals and people with NAFLD, without changes in fasting blood glucose and insulin levels, after spirulina supplementation [18,22]; three studies showed significant decreases in fasting blood glucose and postprandial blood glucose levels in people with type 2 diabetes [33,34,35], and decreased HbA1c [33]. The possible beneficial effects of spirulina consumption on lowering blood glucose concentrations may be due to the fact that spirulina has been suggested to act as an insulin-like protein, or to stimulate the pancreatic β-cells by increasing insulin production and decreasing blood glucose concentrations [28], possibly in a concentration dependent manner as has been shown in animal models [36]. The contradictory results regarding the effects of spirulina supplementation on the indices of glycemic control are evident in systematic reviews and meta-analyses. The results from a recent systematic review and meta-analysis of seven randomized clinical trials (RCTs) (*n* = 338) reported that a daily dose of 8 g of spirulina supplementation decreased significantly plasma triglyceride levels in adults with obesity or diabetes mellitus, but not fasting or postprandial blood glucose levels or HbA1c [28]. Another recent systematic review and meta-analysis of eight RCTs (nine arms) in people with type 2 diabetes reported a significant reduction in fasting blood glucose levels, but no effect on HbA1c or postprandial blood glucose fluctuations after spirulina supplementation in doses of 0.8 to 8 g/day and duration from 45 to 90 days [37]. Another recent systematic review and meta-analysis of seven clinical trials in humans and 27 animal studies reported that spirulina supplementation decreased significantly fasting blood glucose levels in people with type 2 diabetes, and fasting blood glucose levels and HbA1c in diabetic animals, but without changes in body weight, fasting insulin levels, and insulin resistance [38]. Sub-group analyses showed that short-term trials with a duration of less than 2 months and the consumption of a spirulina dose of less than 2 g showed significant improvements in terms of lowering fasting blood glucose in humans [38]. Another systematic review and meta-analysis of 12 RCTs reported that participants taking 2 or more g spirulina/day for 12 or more weeks had significantly lower triglyceride and fasting blood glucose levels, HbA1c, and SBP/DBP [39]. In contrast, another systematic review and meta-analysis reported a significant reduction in fasting blood glucose levels following spirulina supplementation; however, this was observed only in populations with type 2 diabetes older than 45 years, overweight or obese, and those that used spirulina in dosages of 4 or lower g/day [40]. Interestingly, and contrary to fasting blood glucose, this meta-analysis also reported a significant increase in HbA1c concentrations after spirulina supplementation in studies that used less than 4 g/day and those with a duration less than 12 weeks; whereas a significant reduction in HbA1c levels was observed only in studies using spirulina in doses of 4 or more g/day, that lasted 12 or more weeks, and had a sample size of fewer than 40 participants [40]. In addition, pooled analysis from three studies showed a significant reduction in insulin concentrations after intake of spirulina supplements, without data on spirulina dosage [40].

A decrease in glycated hemoglobin A1c (HbA1c) has been suggested to be expected with spirulina consumption or supplementation due to the high iron content of spirulina that could increase hemoglobin levels lowering consequently blood glucose concentrations [41]. It has been suggested that the blood glucose lowering effects may be due to spirulina’s fiber content, protein, and bioactive polypeptides produced after digestion, which may increase insulin secretion and lower glucose absorption [42] and postprandial blood glucose levels even in patients with type 2 diabetes [42,43]. Spirulina consumption has been reported to improve C-peptide and plasma insulin levels [44]. Increased glucokinase activity in the liver cells of rats treated with spirulina suggested increased glucose uptake from the circulation [44]. Spirulina produced high levels of NADP+, resulting in reduced lipogenesis, less oxidative stress, and lowering resistance levels to diabetes [44]. A study in rats with type 2 diabetes examining the effects of ethanol extract and butanol fraction of spirulina on insulin release and glucose homeostasis reported that the ethanol extract and butanol fraction stimulated insulin release from the pancreatic β-cells in a concentration dependent manner [36]. The butanol fraction also similarly stimulated insulin release from perfused rat pancreas by inhibiting dipeptidyl peptidase-4 (DPP-4) activity [36]. This is an important effect of spirulina since DPP-4 is a cytokine secreted from the adipocytes, and has been shown to suppress insulin signaling and induce insulin resistance in muscle in vitro; furthermore, its plasma levels are increased in obesity and correlate positively with all parameters of the metabolic syndrome [45]. Spirulina was also reported to significantly decrease postprandial hyperglycemia after oral sucrose load and increase unabsorbed sucrose content throughout the gut [36]. During in situ intestinal perfusion with glucose, the butanol fraction reduced glucose absorption and promoted gut motility [36]. Moreover, in rats with type 2 diabetes, chronic oral administration of butanol fraction for 28 days significantly decreased blood glucose, increased plasma insulin, pancreatic insulin stored, liver glycogen, and improved lipid profile [36]. Lastly, a study using insulin resistant-HepG2 cell model, reported that spirulina protein had anti-diabetes effects in insulin resistant cell models, with 11 anti-diabetes peptides being identified, and LRSELAAWSR displaying the best activities on DPP-4 [46].

Regarding BP, the results from the two current investigations showed that only the dose of 8 g spirulina as beverage lowered significantly SBP, but not DBP, acutely in 120 min posttest beverage consumption. Our results are in disagreement with one trial showing no BP effects in overweight or obese elderly after 12 weeks of 8 g spirulina supplementation [15], and one trial showing significant decrease only in DBP in people with type 2 diabetes after 12 weeks of 8 g spirulina supplementation [16]. Our results regarding lower doses are in partial agreement with two trials showing no BP effects in people with NAFLD, after 2 g or 6 g of spirulina supplementation [17,18]. Our results are in partial agreement with two trials showing significant decreases in SBP and DBP in overweight individuals after 6 weeks of 4.5 g spirulina supplementation [19], and another trial showing significant decreases in SBP, but not DBP, in people with systemic arterial hypertension after 12 weeks of 4.5 g spirulina supplementation [21]. Our results are also in partial agreement with a trial showing significant decreases in SBP, but not DBP, in people with hypertension and overweight without other cardiovascular disease risk factors after 3 months of 2 g spirulina supplementation [20]. It has been reported that the BP beneficial changes are observed in both sexes, and that youngest people were more BP responsive to spirulina than the other age groups [19]. A diet supplemented with spirulina has been suggested to prevent the synthesis and release of vasoconstricting metabolites to arachidonic acid induced by fructose and attenuated tension development in response to phenylephrine [47]. Vasoconstriction is a fundamental mechanism contributing to the development of atherosclerosis [48]. Others have also shown increased spirulina-induced nitric oxide endothelium synthesis and release, a well-known vasodilator metabolite [49]. Moreover, it has been reported that the C-phycocyanin content in spirulina inhibited platelet aggregation through inhibition of calcium mobilization and mediation of free radicals released by platelet [50]. It has also been proposed that high potassium and low sodium contents of spirulina may have positive effects on BP. A pooled data analysis reported that spirulina consumption was associated with a significant decrease in DBP [39]. In an ex vivo vessel model, the peptide fraction isolated from spirulina showed direct endothelium-dependent vasodilation. This mechanism was proposed to be through a phosphoinositide-3-kinase (PI3K)/AKT (serine/threonine kinase AKT) pathway that converges on nitric oxide release [51]. The peptide induced a significant reduction in BP in vivo [51]. Substances, such as the tripeptide lle-Gln-Pro (IQP) present in spirulina also showed antihypertensive activity by inhibiting angiotensin converting enzymes [52]. A meta-analysis of 5 RCTs that included 230 people to examine spirulina’s effects on BP as supplement, in doses from 1 to 8 g spirulina/day, for 2 to 12 weeks, reported that spirulina decreased SBP by about 4.6 mmHg and DBP by 7 mmHg [53], which is in partial agreement with our results.

Results from our current investigations showed no changes in acute appetite scores after consumption of different doses of spirulina. Our findings disagree with two other studies using 1–2 g spirulina supplementation for 12 weeks in overweight or obese individuals showing significantly lower appetite scores after spirulina supplementation [22,23]. The lack of reported beneficial effects on appetite scores in our current investigations may be due to the short duration of the trials, which should be further investigated.

### 4.1. Studies Limitations and Advantages

The strength of our studies includes the randomized, crossover, double-blind design where each participant served as his/her own control. The major limitation of the current investigations is the acute feeding protocol, which does not allow conclusions for long-term benefits. Another limitation may be the small sample size, although the results were consistent with low diversity. Moreover, our studies were conducted in healthy, normal body weight, normoglycemic, and normotensive young adults and, therefore, our results need to be confirmed in other populations i.e., middle-aged, or elderly people with prediabetes or type 2 diabetes with and without obesity. Furthermore, our results need to be confirmed in RCTs of longer duration.

### 4.2. Practical Applications

To the best of our knowledge, these trials determined for the first time the effects of different doses of spirulina consumed as food and not as supplement, on glycemic responses. Our results proposed the dose of 8 g of spirulina as a potentially optimal dose for ameliorated postprandial blood glucose concentrations and SBP, which needs to be confirmed in longer duration studies and in high cardiometabolic risk populations. The assessment of glycemic and BP effects of spirulina added in foods and regarded as a functional food/ ingredient, may be considered a crucial element for an effective primary and secondary CVD prevention.

## 5. Conclusions

In conclusion, the results from the current investigations showed that spirulina led to ameliorated glycemic responses and SBP, indicating that its consumption may be a healthy dietary alternative for glycemic control and hypertension. Long-term studies with a larger sample size are needed to provide an insight into the mechanisms by which a fortified food system with spirulina can improve the glycemic responses of people with insulin resistance and high cardiometabolic risk.

## Figures and Tables

**Table 1:** Nutrient analysis per 100 g of cookie and spirulina cookie.

	Cookie (C)	Spirulina Cookie (CS)
Energy (kcal)	443.0 kcal/1853.5 kJ	439.5 kcal/1838.9 kJ
Fat (g)	19.9	20.2
Saturated fat (g)	9.7	9.9
Cholesterol (mg)	5.5	5.5
Carbohydrates (g)	59.6	57.2
Dietary fiber (g)	1.2	1.3
Sugar (g)	25.0	25.2
Protein (g)	6.04	8.18
Sodium (mg)	101.2	148.8
Potassium (mg)	124.3	181.4

Proteins were assessed by AOAC 920.87, 2005; total fat and saturated fat by AOAC 996.06; available carbohydrates, sugars, and dietary fibers by the Megazyme assay kit (Megazyme kit-K-ACHDF, Megazyme Ltd., Scotland, UK), moisture by AOAC 930.15.

**Table 2:** Ingredient of cookie and spirulina cookie per 100 g.

	Cookie (C)	Spirulina Cookie (CS)
All-purpose flour	45.5 g	41.0 g
Spirulina powder	-	4.5 g
Sugar	22.5 g	22.5 g
Milk powder	5.7 g	5.7 g
Vanilla	0.1 g	0.1 g
Baking powder	0.6 g	0.6 g
Plant-based butter	25.6 g	25.6 g

**Table 3:** Nutrient composition of spirulina powder per 100 g based on the food label.

	Spirulina Powder
Energy (kcal)	290 kcal/1213 kJ
Protein (g)	57.47
Fat (g)	7.72
Saturated fat (g)	2.65
Carbohydrates (g)	23.9
Dietary fiber (g)	3.6
Sugars (g)	3.1
Sodium (mg)	2.62

Proteins were assessed by AOAC 920.87, 2005; total fat and saturated fat by AOAC 996.06; available carbohydrates, sugars, and dietary fibers by the Megazyme assay kit (Megazyme kit-K-ACHDF, Megazyme Ltd., Scotland, UK), moisture by AOAC 930.15.

**Table 4:** Participants’ baseline characteristics in Trials 1 and 2.

	Trial 1	Trial 2
Characteristics	Total	
N	13 (4 men, 9 women)	13 (3 men, 10 women)
Age (years)	25.2 ± 1.0	23.8 ± 0.7
Weight (kg)	65.1 ± 4.8	69.4 ± 3.8
Height (cm)	168 ± 0.03	169 ± 0.02
Body mass index (BMI; kg/m^2^)	22.8 ± 0.9	24.1 ± 1.0
Body fat (kg)	15.6 ± 1.8	18.3 ± 2.1
Muscle mass (kg)	27.6 ± 2.6	28.5 ± 1.3
Waist circumference (cm)	79.1 ± 2.72	79.5 ± 3.8
Hip circumference (cm)	99.4 ± 1.8	100.3 ± 2.8
**Dietary intake (from 24-h recall)**		
Protein (g)	60.87 ± 5.43	73.15 ± 6.70
Carbohydrate (g)	193.98 ± 19.91	193.51 ± 20.15
Fat (g)	60.25 ± 5.89	59.61 ± 4.73
Saturated fat (g)	20.27 ± 2.43	23.88 ± 2.68
Total cholesterol (g)	215.44 ± 29.00	259.40 ± 27.71
Fiber (g)	17.26 ± 2.04	27.79 ± 8.92
Sodium (mg)	2031.56 ± 242.82	2450.43 ± 201.12
Energy intake (kcal)	1561.65 ± 133.40	1613.85 ± 150.06

Values are means ± SEM.

**Table 5:** Incremental area under the curve (iAUC) for blood glucose, glycemic index (GI), glycemic load (GL), and peak for blood glucose values of cookie without spirulina (C), cookie with spirulina (CS), and white bread, relative to the reference food D-glucose.

Meal (Serving Portion Containing 50 g Available Carbohydrates)	iAUC (mg·120 min·dL^−1^)	GI (D-Glucose as Reference Food)	GL (D-Glucose as Reference Food)	Blood Glucose Peak Value (mg/dL)
D-Glucose	4848 ± 508 ^a^	100 ^a^	-	77 ± 6 ^a^
White bread	3646 ± 400 ^b^	73 ± 6 ^b^	36 ± 3 ^b^	53 ± 5 ^b^
C (76.88 g)	2987 ± 341 ^b^	59 ± 5 ^b^	22 ± 2 ^c^	47 ± 5 ^b^
CS (80.16 g)	3022 ± 389 ^b^	60 ± 6 ^b^	21 ± 2 ^c^	46 ± 4 ^b^

Data are means ± SEM. Each value represents the mean thirteen participants. Values labeled with different superscript letter are significantly different (*p* < 0.05). Means were compared column-wise by using one-way ANOVA for factor “treatment”, period, and sequence of treatment, and post hoc Tukey test with Bonferroni correction to account for multiple comparisons between test meals; *p*-values < 0.05 were accounted as significant.

**Table 6:** Multi-level random effects regression model for differences of mean glucose levels at 120′ from the baseline (Δ120–0).

Δ120–0	Coef.	Std. Err.	P > z	[95% Confidence Interval]	
Dose 4 g Spir	−8.877058	3.693365	0.016	−16.11592	−1.638196
Dose 6 g Spir	−4.301632	4.121417	0.297	−12.37946	3.776197
Dose 8 g Spir	−8.550541	3.733738	0.022	−15.86853	−1.23255
cons	−2.450879	3.650722	0.502	−9.606162	4.704404
sigma_u	6.110511	1.896142		3.326157	11.22567
sigma_e	9.502128	1.03656		7.67301	11.76728
rho	0.2925543	0.1449218		0.0849786	0.6104398

Time/Visit when treatment was received was included in the model and had no significant effect. All values depicted are compared to control meal (50 gr D-Glucose with 0 gr Spirulina). Abbreviations: Δ120–0: Mean glucose levels at 120′ difference from the baseline after consumption of beverages with 4, 6, 8 g spirulina; Spir = spirulina; cons; sigma_u; sigma_e; rho; P = *p*-value.

**Table 7:** Multi-level random effects regression model for area under the curve for blood glucose concentrations from 90 to 120 min (AUC90–120).

AUC90–120	Coefficient	P > z	[95% Confidence Interval]	
Dose 4 g Spirulina	−143.7275	0.053	−289.5495	2.094498
Dose 6 g Spirulina	−111.7423	0.156	−265.9917	42.50708
Dose 8 g Spirulina	−166.368	0.026	−312.9799	−19.75609
cons	347.1318	0	190.1622	504.1015
sigma_u	151.2978		89.32215	256.2748
sigma_e	192.2347		155.2247	238.0691
rho	0.3825037		0.1486562	0.6716199

Time/Visit when treatment was received was included in the model and had no significant effect. All values depicted are compared to control meal (50 gr D-glucose with 0 gr spirulina). Abbreviations: AUC 90–120: Area under the curve for blood glucose from 90 to 120 min after consumption of beverages with 4 g, 6 g, and 8 g spirulina; cons; sigma_u; sigma_e; rho; P = *p*-value.

## Data Availability

Not applicable.
